# Review of Graphene Growth From a Solid Carbon Source by Pulsed Laser Deposition (PLD)

**DOI:** 10.3389/fchem.2018.00572

**Published:** 2018-11-21

**Authors:** Yannick Bleu, Florent Bourquard, Teddy Tite, Anne-Sophie Loir, Chirandjeevi Maddi, Christophe Donnet, Florence Garrelie

**Affiliations:** Laboratoire Hubert Curien UMR 5516 CNRS, Université Jean Monnet, University of Lyon, Saint-Étienne, France

**Keywords:** graphene, 2D materials, doped graphene, pulse laser ablation deposition, sensors

## Abstract

Graphene is a remarkable two-dimensional (2D) material that is of great interest to both academia and industry. It has outstanding electrical and thermal conductivity and good mechanical behavior with promising applications in electronic devices, supercapacitors, batteries, composite materials, flexible transparent displays, solar cells, and sensors. Several methods have been used to produce either pristine graphene or doped graphene. These include chemical vapor deposition (CVD), mechanical exfoliation, decomposition of SiC, liquid-phase exfoliation, pulsed laser deposition (PLD). Among these methods, PLD, which is routinely used for growing complex oxide thin films has proved to be an alternative to the more widely reported CVD method for producing graphene thin films, because of its advantages. Here we review the synthesis of graphene using PLD. We describe recent progress in preparing pristine graphene and doped graphene by PLD, including deposition processes and characterization. The goal of this complete survey is to describe the advantages of using the technique for graphene growth. The review will also help researchers to better understand graphene synthesis using the PLD technique.

## Introduction

Graphene is considered to be an emergent 2D material for modern science because of its unique versatile properties including high conductivity, transparency, strength, and thermal conductivity with many potential applications in research and industry as transparent electrodes, field emitters, biosensors, batteries, composites, and so on (Geim and Novoselov, 2007; Lee et al., 2008; Bonaccorso et al., 2010; Kuila et al., 2011; Novoselov et al., 2012; Kim et al., 2013). Various techniques exist for the production of graphene including chemical vapor deposition (CVD), physical vapor deposition (PVD), chemical synthesis, micromechanical exfoliation, epitaxial growth on SiC or metal substrates (Blake et al., 2007; Stankovich et al., 2007; Faugeras et al., 2008; Kim et al., 2009; Lambert et al., 2010; Wang et al., 2011; Shen et al., 2012; Tatarova et al., 2013; Fortgang et al., 2016; Tite et al., 2016). Each method has its own advantages and drawbacks. For example, mechanical exfoliation does not require high processing temperatures, but is labor intensive and the lack of uniformity and the presence of residues limit the mobility of the exfoliated layers, both of which are serious drawbacks. High temperature processes are needed to remove the residues (Choi et al., 2010). Although mechanical exfoliation produces high quality graphene film, the graphene films can only be produced in small sizes (usually ≪ 1000 μm2) (Xu et al., 2014) and is consequently not suitable for scaling up and for industrial production. Though large-area graphene can be achieved by epitaxial growth on SiC, synthesis and the subsequent transfer (Xu et al., 2014) of graphene to the desired substrates using SiC is very expensive and is only suitable for certain electronic devices. The CVD method can also produce large-scale graphene on some transition metals, but here again, removal of undesirable catalysts and transfer of graphene to suitable substrates limit the range of applicability (Li et al., 2009; Park and Ruoff, 2009). Among all the techniques, PLD method is known to be an appropriate technique for thin film growth, mostly without any changes in the composition of the target because ablation occurs as soon as the target is irradiated with a laser beam. In addition to synthesis of based carbon film (Sikora et al., 2010b), by co-ablation, PLD makes it possible to incorporate any dopant or addition elements at any concentrations (Garrelie et al., 2008; Sikora et al., 2009; Maddi et al., 2016), i.e., a way to obtain doped or alloyed carbon films.

Until now, there has been no review of the PLD technique for manufacturing pristine and doped graphene. This review provides an overview of recent progress, efforts, and challenges in the production of both pristine and doped graphene using the PLD method. We begin with a brief presentation of this remarkable graphene material and of the PLD technique. Next, the core section concerning progress in synthetizing graphene using PLD is divided into two parts: part one on graphene synthesis by PLD without a catalytic metal layer and part two on graphene growth by PLD using a metal catalyst. Lastly, we present some applications for graphene obtained using the PLD technique and some suggestions for further research. The review will give researchers a better understanding of the graphene production using the PLD route.

## Brief presentation of graphene

Carbon is an important material for life and all organic chemistry sources. Graphene is one of the allotropes of carbon and is considered as the starting point for recognition of the electronic properties of others (Wallace, 1947; Geim, 2012).

In the early stages of research, it was assumed graphene would not be found in the free state (Wan et al., 2012). In addition, it is unstable regarding warped structures including fullerenes and nanotubes. Luckily, in 2004, the theoretical concepts concerning this material became real, when free-standing graphene was found to exist and especially when experiments confirmed that its charge carriers were Dirac fermions without any mass (Novoselov et al., 2005; Garg et al., 2014). For their innovative experiments on graphene material, Geim and Novoselov were awarded the Nobel Prize in Physics 2010. Graphene exhibits a 2D honeycomb lattice, with a compact single layer of carbon atoms.

**Figure 1:**
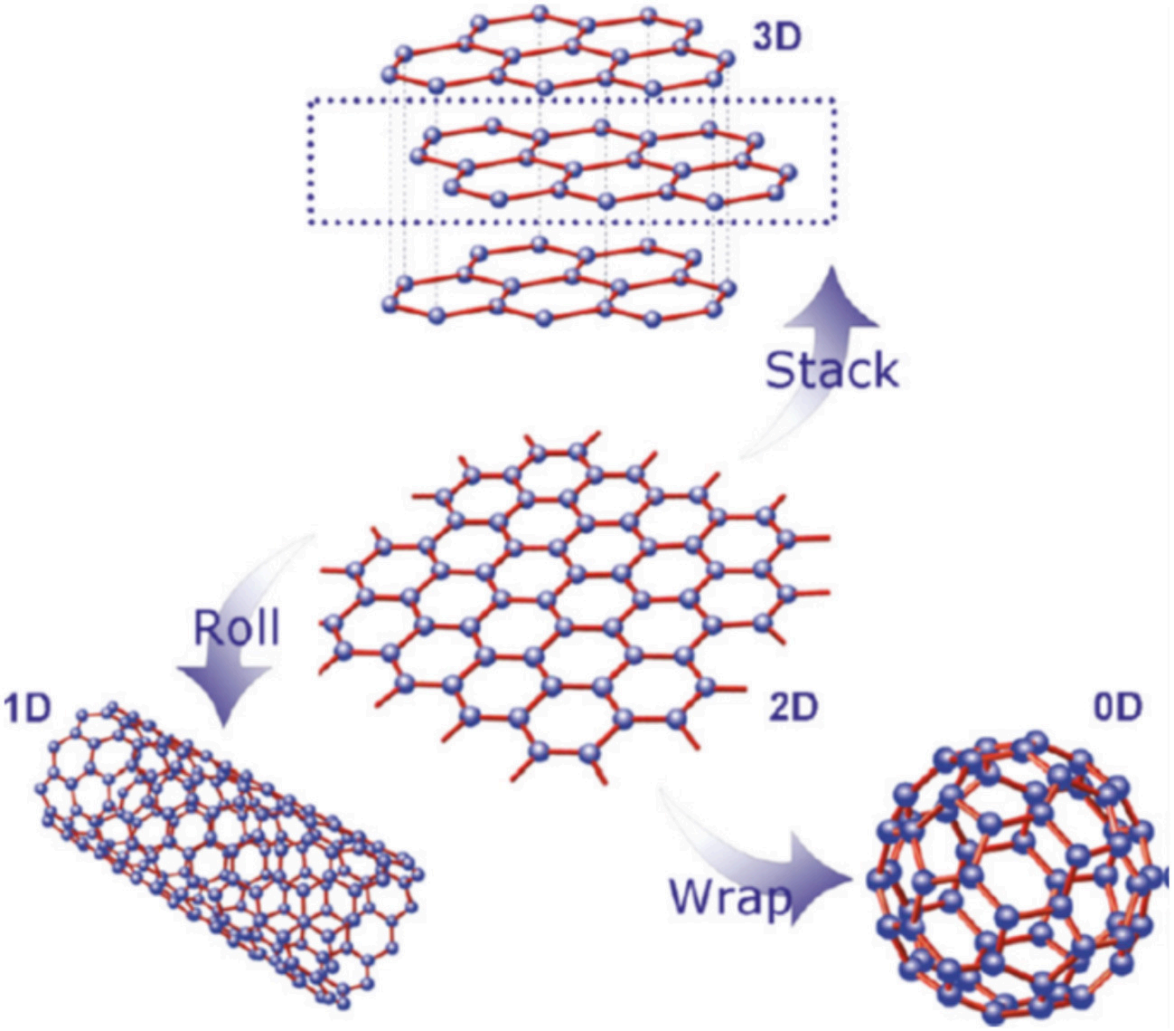
The graphene building block for various carbon allotropes [Reproduced from Wan et al. (2012) with permission of American Chemical Society].

**Figure 2:**
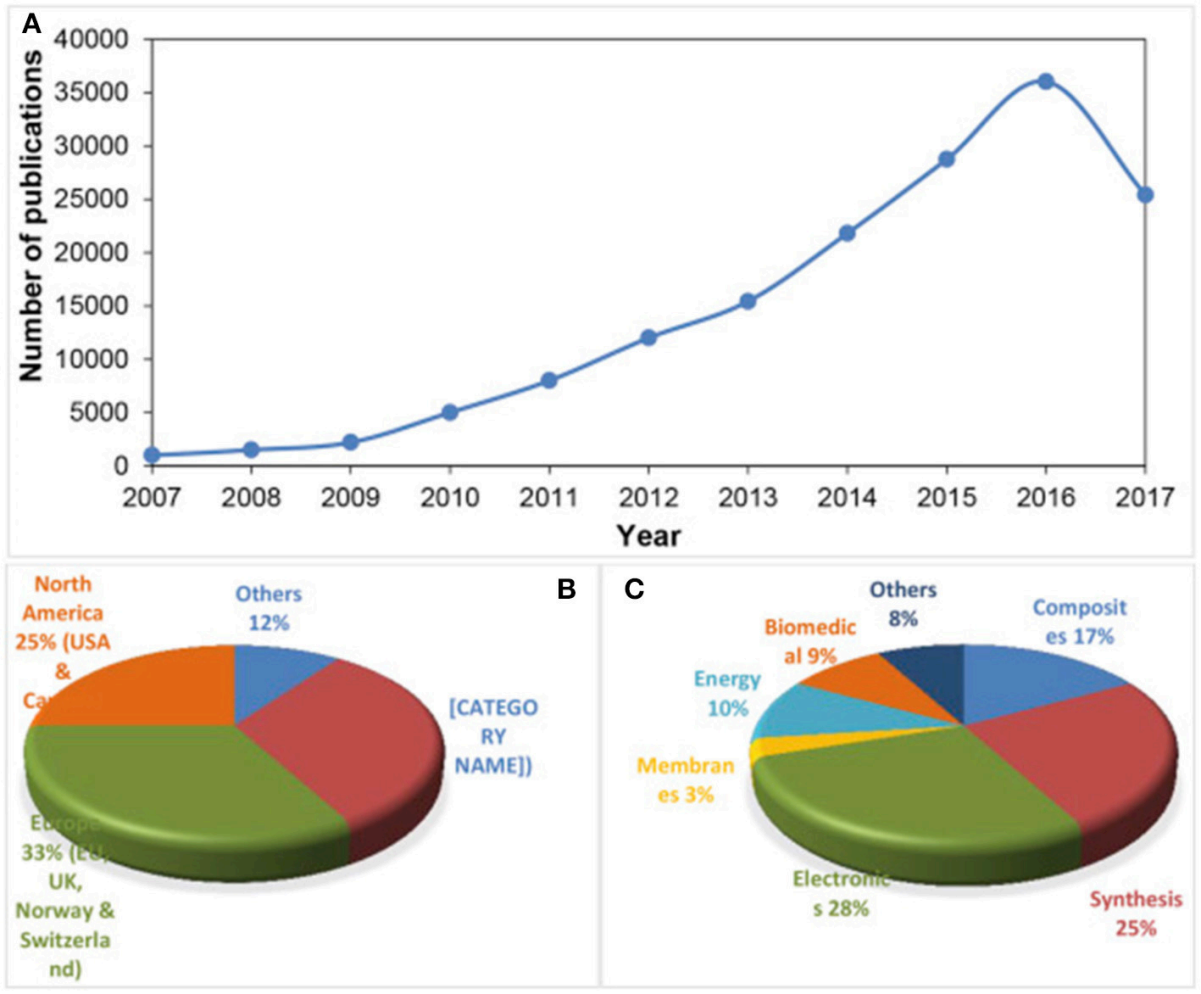
**(A)** Publications on graphene from 2007 to August 2017, and expected to reach at least 40,000–45,000 publication by the end of 2017 [Source: Web of Science], **(B)** proportion of total publications per country and **(C)** per sector.

Being the basic block for all graphitic materials, graphene plane can be wrapped into 0D fullerenes, rolled into 1D nanotubes or stacked into 3D graphite (Zhang et al., 2005; Geim and Novoselov, 2007; Geim, 2012) as shown in Figure 1. Graphene can be grown on top of substrates (Fortgang et al., 2016; Tite et al., 2016), synthetized in liquid suspension (Geim, 2012) or as suspended membranes (Narayan et al., 2016). Thanks to its remarkable properties, graphene has revolutionized physics of condensed matter and more generally nanoscience. As a possible substitute of silicon in electronics and other advanced technologies, as well as, being a potential rival for indium tin oxide (ITO) as transparent conducting electrode, graphene has been the subject of huge interest by many research teams worldwide and as a result, there has been a considerable increase in literature on the topic (Figure 2).

### Graphene structure

The word “graphene” is made up of the prefix “graph” from graphite and the suffix “ene” from the carbon/carbon double bonds (Bianco et al., 2013). It also refers to a single layer of graphite, with sp^2^-hybridized carbon atoms forming a hexagonal lattice with partially filled π-orbitals above and below the plane of the sheet. The term “graphene” is often prefixed by “monolayer,” “bilayer,” “few-layer” or “multi-layer.” This classification was introduced because the electronic properties of bi-, few-layer, and multi-layer graphene are distinct from the electronic properties of graphite (Geim and Novoselov, 2007; Bianco et al., 2013).

While the existence of monolayer graphene in a rippled form with no stacking of sheets is generally accepted, 4–6 few-layer graphene can display different stacking arrangements, including mainly the Bernal stacking (ABAB), the rhombohedral stacking (ABCABC) (Partoens and Peeters, 2006; Mak et al., 2010). Turbostratic graphene, with an interlayer spacing >0.342 nm larger than that of crystalline graphene (0.335 nm) (Horiuchi et al., 2003), is a specific lattice arrangement with no discernible stacking order. Rotation and translation of the graphene sheets are possible, due to the increased inter-planar distance between graphene planes, due to weaker inter-planar bonding.

**Figure 3:**
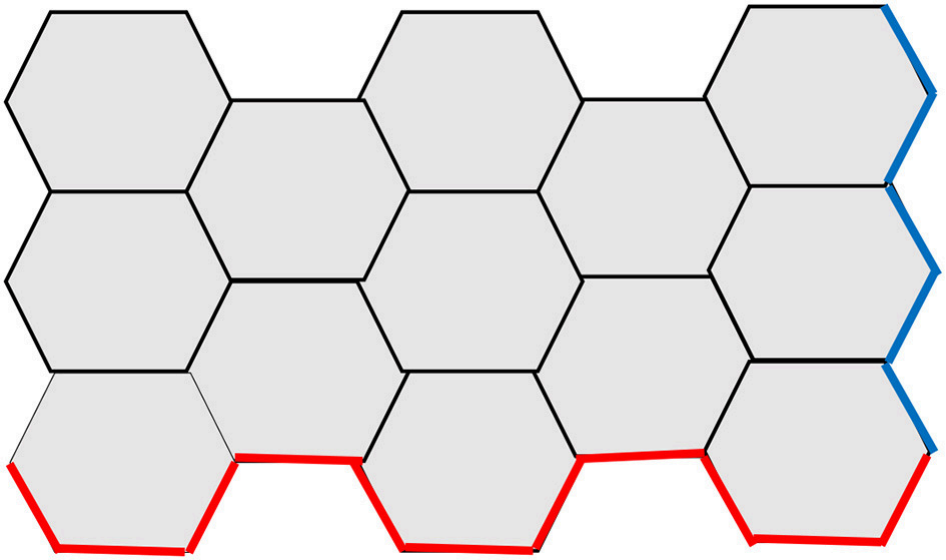
Armchair (red) and zigzag (blue) edges in monolayer graphene.

Another structural parameter that can influence the graphene properties is its edges. Graphene edges can exhibit armchair or zigzag configurations (Figure 3), with different electronic and magnetic properties (Malard et al., 2009; Jia et al., 2011; Acik and Chabal, 2012). Research into the synthesis of graphene structures with defined edges currently aims at tuning its properties for specific applications (Rao et al., 2009).

### Graphene properties and applications

Graphene has exceptional properties, it is described as the thinnest, most flexible and strongest material known (Edwards and Coleman, 2013), it is impermeable to gases (Bunch et al., 2008). Graphene has C-C bond length of about 0.142 nm, with a weak Van der Waals interaction between layers. Table 1 lists some of the outstanding properties of single layer graphene so far.

**Table 1:** The most exceptional properties of single layer graphene.

**Properties**	**Values**	**References**
Optical transparency	97.7%	Nair et al., 2008
Electron mobility	200,000 cm2 v^−1^ s^−1^	Bolotin et al., 2008
RT Thermal conductivity	5,000 W m^−1^ K^−1^	Balandin et al., 2008
Specific surface area	2,630 m2g^−1^	Edwards and Coleman, 2013
Breaking strength	42 N.m^−1^	Hsu, 2009
Elastic modulus	1 TPa	Lee et al., 2008
Fermi velocity	1 × 10^6^ m s^−1^	Du et al., 2008

**Figure 4:**
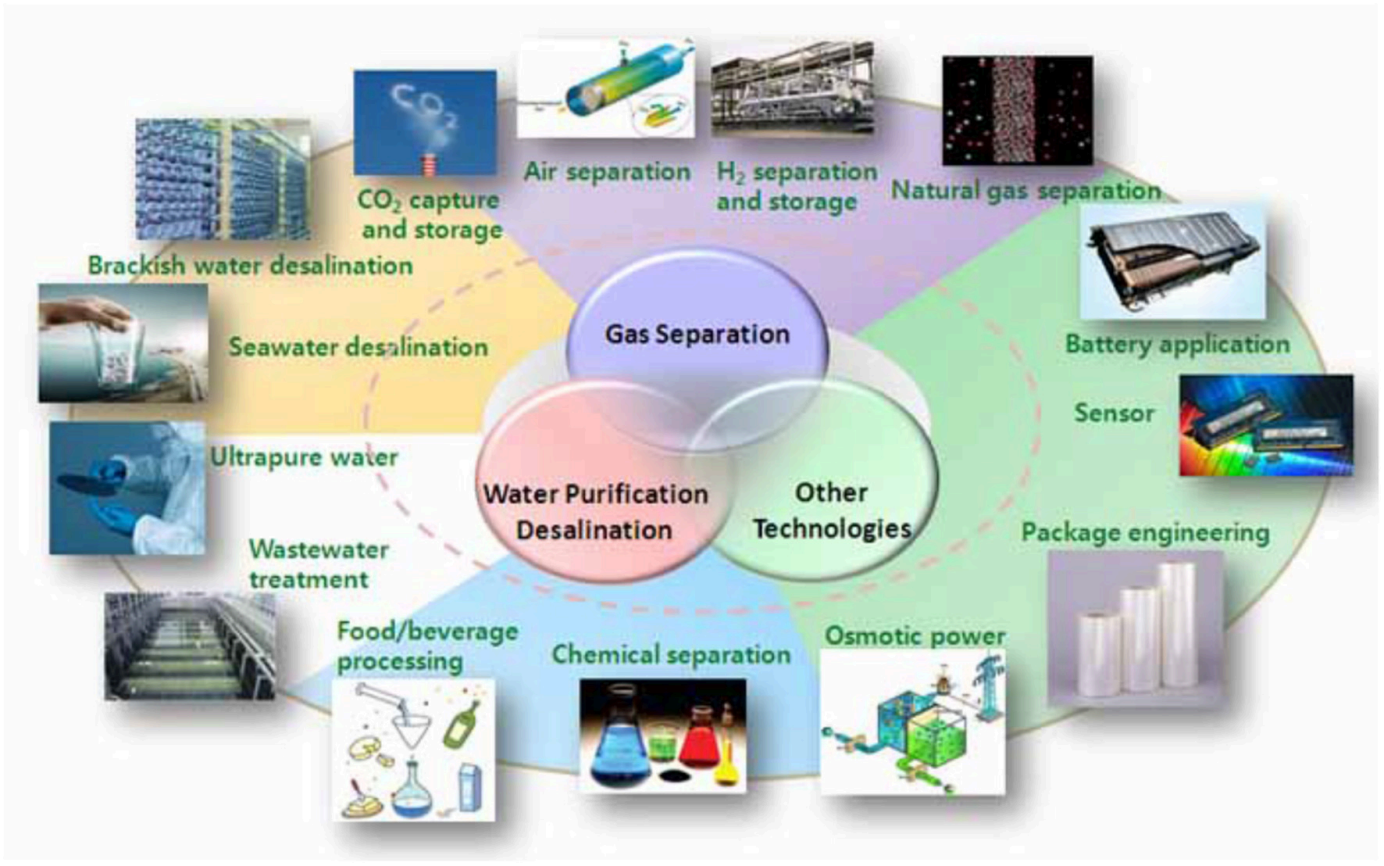
Overview of graphene applications in various sectors (from https://www.pgmcapital.com/why-investing-in-graphene-can-be-lucrative/).

All the exceptional properties of graphene have led to real applications. For example, graphene-based materials can be used as semi-conductors. Figure 4 shows different applications of graphene in various industrial sectors. Enoki and Kobayashi (2005) examined the exclusive magnetic properties of graphene in electronic and magnetic applications, such as spin glasses or magnetic switch.

**Figure 5:**
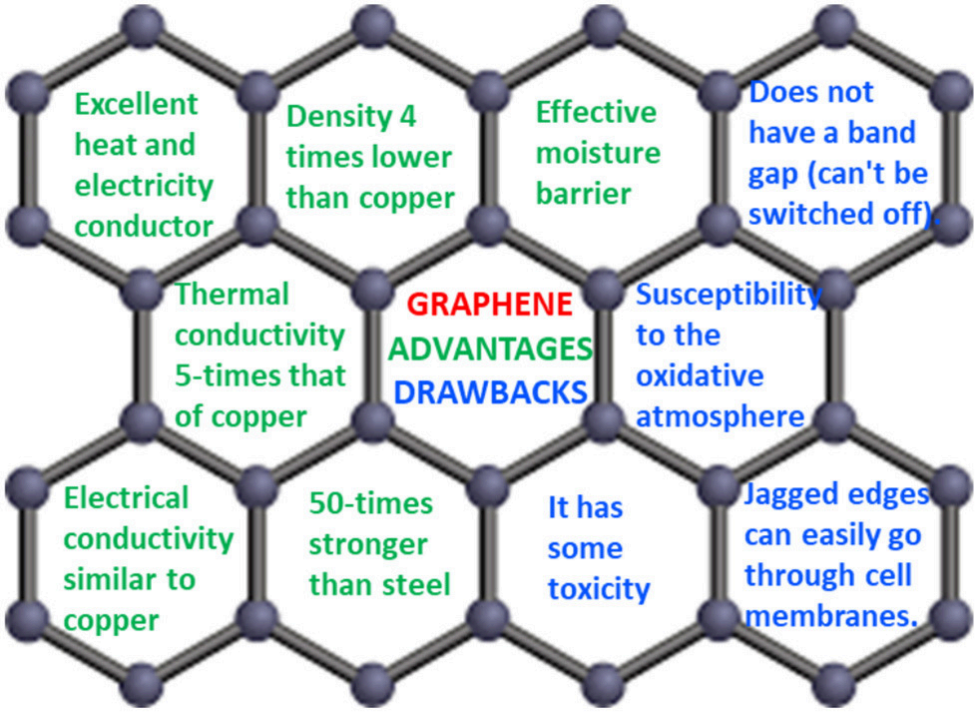
Main advantages and drawbacks of graphene reported in references (Allen et al., 2010; Skulason et al., 2010).

The main advantages and drawbacks of graphene are reported in Figure 5. It is worth noting that most properties are recorded on high quality monolayer graphene deposited on small areas, and may not be possible using larger and/or few-layer graphene sheets. One example is the decrease in the surface area of graphene and the increase in its transparency, from monolayer to few-layer graphene (Allen et al., 2010).

Graphene's physical properties may also be sensitive to the thickness of the layers or to the number of layers. For instance, a gradual change in its electronic properties has been observed when increasing numbers of layers (AlZahrani and Srivastava, 2009; Jana and Ray, 2015). The measured thermal conductivity is highly influenced by the graphene thickness: the value for a four-layer graphene is almost the same as that of bulk graphite (Klintenberg et al., 2009). Hardness and in elastic modulus are strongly dependent on the number of graphene, layers, and a linear decrease in both properties is observed when the number of layers increases up to four (Zhang and Pan, 2012).

## The PLD technique

**Figure 6:**
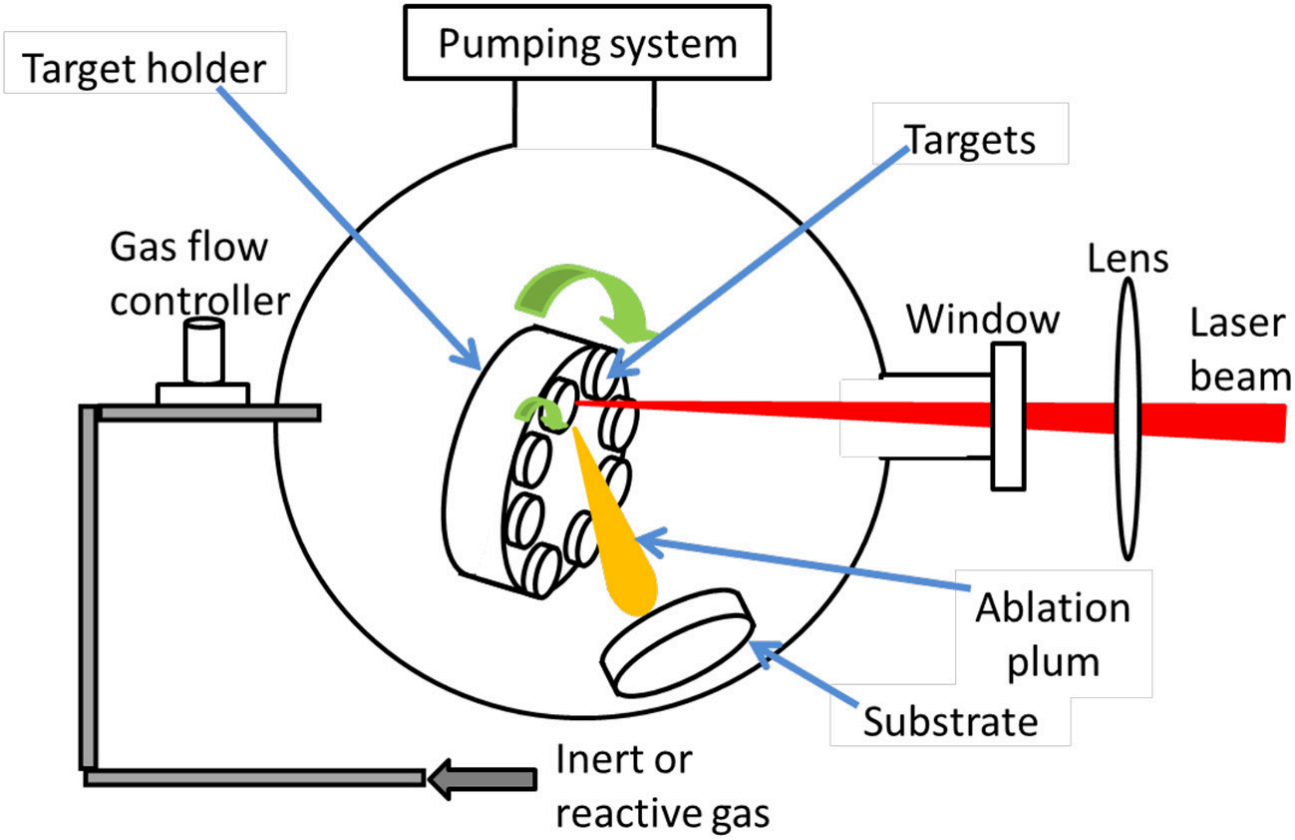
Schematic diagram of a representative laser deposition tool.

The PLD technique was first used by Smith and Turner (1965) in 1965 to manufacture semiconductors and dielectric thin films using a ruby laser, which is considered as a very versatile thin film growth process. Since the laser source is located outside the deposition chamber, PLD deposition can be performed either in ultra-high vacuum or in ambient gas (Krebs et al., 2003). It is possible to deposit all kinds of carbon-based materials, including fullerenes (Ying et al., 1996), carbon nanotubes (Radhakrishnan et al., 2007), graphite, and diamond-like carbon (Sikora et al., 2009, 2010a,b; Acharya et al., 2010). With the PLD technique, the laser ablated species have high kinetic energy up to a few keV, allowing to deposit adherent thin films at relatively low temperatures compared to other techniques. Figure 6 is a schematic diagram of PLD. Inside the vacuum chamber (ultra-high vacuum, UHV), targets of elementary or alloy elements are struck at an angle of 45° by a high energy focused pulsed laser beam.

The species ablated from the target(s) are deposited directly onto the substrate. The principle behind the PLD mechanism can be briefly described as follows. The focused laser beam, striking the surface of a solid target during a short time, induces an energetic plasma plume containing ions and atoms, impinging the substrate in front of the target. Depending on various process parameters, including the substrate temperature, a single-crystal, polycrystalline or amorphous film can be obtained (Nomura et al., 2004, 2006; Maddi et al., 2016). The quality of the deposit can be controlled by adjusting the experimental parameters, mainly the laser parameters (fluence, wavelength, pulse-duration, and repetition rate), and the deposition conditions (target-to-substrate distance, temperature, nature and pressure of the environment, etc.) (Koh et al., 2010; Qian et al., 2011; Wang et al., 2011).

Compared to CVD, the PLD method is conceptually simple, versatile, rapid, and cost effective, enables good control of thickness and morphology, requires only a low temperature for growth, and can be used with temperature sensitive materials, especially those with an active chemical surface. In addition, composite thin films with complex composition can be deposited by using several targets to perform co-ablation in a controlled and reproducible way. Another difference between the CVD and PLD is the carbon source. In CVD, the carbon source is a gaseous gas mixture, whereas PLD requires a solid carbon target, thus limiting the carbon source during segregation to that supplied during target ablation. Lastly, the PLD energetic source of carbon allows ablated species to penetrate deep into the substrate surface, rather than remaining on the film surface (Koh et al., 2012).

In comparison with other PVD methods, including thermal evaporation or sputtering, PLD has two main specificities. Firstly, synthesis is performed by a pulsed mode, meaning that a small amount of matter can be grown in a few microseconds. Secondly, because of the rapid intense heating of the target, stoichiometric growth can be readily achieved using PLD (Yang and Hao, 2016).

In the context of graphene growth, PLD provides an alternative way to control the thickness and composition of the graphene precursor, by laser wavelength and power, to that of CVD, where control is achieved by temperature and gas pressure (Hemani et al., 2013). PLD was used for the first time by Cappelli et al. (2005) for graphene synthesis in 2005. Their PLD depositions were performed on Si <100> substrates, at temperatures ranging from room temperature (RT) to 900°C using Nd: YAG laser operating in the near IR (λ = 532 nm, repetition rate ν = 10 Hz, pulse width τ = 7 ns, fluence φ ~7 J/cm^2^, deposition time = 15 min). Since then, many others groups have used PLD to synthesize graphene. Tables 2, 3 give an overview of graphene fabrication using this versatile method up to now. Table 1 lists PLD techniques without metal catalysts and Table 2 shows the PLD techniques using metal catalysts. In fact, some groups certainly used the PLD graphene growth method without catalysts to avoid the transfer process. Other groups preferred to use metal catalysts to improve the quality of the graphene. In the following section, we describe the two different ways to grow graphene using the PLD technique.

**Table 2:** Summary of graphene grown on different substrates using the PLD technique without a catalytic layer.

	**Laser parameters**	**Atmosphere**	**Deposition conditions**	**Targets**	**Distance Target/Substrate**	**Graphene types**	**References**
Graphene/Si <100>	Nd:YAG, λ = 532 nm, τ = 7 ns, ν = 10 Hz, fluence = 7J/cm2	10^−5^ Pa	RT and 900°C/15min	HOPG	5 cm	Nano sized graphene clusters	Cappelli et al., 2005
Graphene/Si <100>	Nd:YAG, λ = 1,064 nm, τ = 7 ns, ν = 10 Hz, fluences = 7.8, 11 et 14 J/cm2	10^−4^ Pa	RT to 800°C	Graphite	N/A	Well-ordered nanographene	Cappelli et al., 2007
Graphene/Si	Nd:YAG, λ = 532 nm, τ = 7 ns, ν = 1 Hz, fluences = 0.8–20 J/cm2	10^−5^ Torr—grown in 1 Torr argon gas	RT	HOPG	15–60 mm	Freestanding 2D few-layer	Qian et al., 2011
Graphene/SiO_2_/Si, Graphene/SiNx/Si Graphene/p-Si	KrF, λ = 248 nm, τ = 20 ns, ν = 10 H, fluences = 3, 5, and 6 J/cm2	10^−8^ mbar—grown in 20 mTorr Ar/O_2_ gas	300, 593, and 973 K	HOPG	15–60 mm	Nanostructured graphene	Sarath Kumar and Alshareef, 2013
Graphene/SiNx/SiNGraphene/SiNx/Si	KrF laser, λ = 248 nm, *t* = 20 ns, ν = 10 Hz, fluence = 6,1 J/cm2	Gr is grown in 20 mTorr ArN-Gr is grown in 20, 100, 250, and 500 mTorr N_2_ gas	973 K	HOPG	N/A	Graphene thin films of both p and n-types	Sarath Kumar et al., 2013
Graphene/SiO_2_	Nd:YAG, fluence = 5 J/cm2	10^−6^ mbar + ambient oxygen during carbon deposition	RT to 800°C/15 min	Graphite	3 cm	Multi-few layer graphene	Kumar and Khare, 2014
Graphene/Quartz Graphene/Sapphire Graphene/n-Si	KrF, λ = 248 nm, τ = 25 ns, ν = 5 Hz, fluence = 4 J/cm2	10^−5^ Pa—grown in 10 Pa Ar gas	750°C/90 s	HOPG	5 mm	Few-layer graphene	Xu et al., 2014
Graphene/Sapphire	Nd:YAG, λ = 266 nm, τ = 20 ns, ν = 10 Hz, fluence = 1,2 J/cm2	10^−5^ Torr	400, 500, and 600 °C/90 s	HOPG	40 mm	Few-layer graphene	Na et al., 2015
Graphene/n-Si(100)	Ti:sapphire fs laser, λ = 800 nm, τ = 80 fs, ν = 1 Hz, fluences = 0.1, 0.3, and 0.5 J/cm2	10^−6^ Torr	300 and 473 K	HOPG	50 mm	Few-layer graphene	Xiangming Dong et al., 2015
Graphene/Cu foil	Nd:YAG, λ = 1064 nm, τ = 6 ns, ν = 5 Hz, laser energy = 50 mJ/pulse	10^−5^ Torr	300, 400, and 500°C	Graphite	5 mm	Few-layer graphene	(Abd Elhamid A. E. M. et al., 2017)
N-Graphene/SiO_2_/Si	KrF laser, λ = 248 nm, τ = 20 ns, ν = 10 Hz, laser energy = 100 mJ	Gr is grown in 10^−5^ Pa N-Gr is grown in 9, 50, 100, 240 Pa of nitrogen gas	1053K	Graphite	5 cm	N-doped graphene	Ren et al., 2017

**Table 3:** Summary of graphene grown on different substrates using the PLD technique with a catalytic layer.

**Stacking order**	**Laser parameters**	**Atmosphere**	**Deposition conditions**	**Targets**	**Distance Target-Substrate**	**Catalytic layer**	**Annealing conditions**	**Type of graphene**	**References**
a-C/Ni/Si	ArF laser, λ = 193 nm, τ = 20–30 ns, ν = 10 Hz, laser energy = 300 mJ	10^−6^ Torr	1100, 1200, 1300°C	Pyrolyticcarbon	X	Ni (500 nm)	1100, 1200, 1300°C before C deposition	Transparent Few-layer	Zhang and Feng, 2010
a-C(7 nm)/Ni/n-Si	KrF, λ = 248 nm, τ = 25 ns, ν = 10 Hz, laser energy = 50 mJ	5.10^−6^ Torr	750°C −1.5 min	Graphite	X	Ni (600 nm)	750°C−1.5 min during C deposition	Few-layer	Koh et al., 2010
a-C(6 nm)/Ni/SiO_2_/Si	KrF, λ = 248 nm, fluence (Ni) = 5.43 J/cm2, fluence 4.40 J/cm2, ν = 4 Hz	2.10^−6^ Torr	RT (Ni deposition) 650°C (C deposition)	Nickel Graphite	35 mm	Ni (25–75nm)	650°C-1 h before C deposition	Few-layer	Wang et al., 2011
a-C/Ni/n-Si	KrF, λ = 248 nm, τ = 25 ns, ν = 10 Hz, laser energy = 50 mJ	5.10^−4^ Pa	750°C −1.5 min	Carbon	X	Ni, Cu, Co, Fe	750°C −1.5 min during C deposition	Few-layer	Koh et al., 2012
a-C/Ni/SiO_2_/Si	KrF, λ = 248 nm, τ = 20 ns, ν = 10 Hz, laser energy = 75 mJ	5–6.10^−6^ Torr	1010°C	Carbon	X	Ni (300 nm)	1010°C during C deposition	Monolayer/Bilayer Few-layer	Hemani et al., 2013
Ni/a-C (20 nm)/n-Si	KrF laser, λ = 248 nm, τ = 20 ns, ν = 10 Hz, fluence = 15 J/cm^2^	10^−4^ Pa	RT- 10 min	Graphite	3.6 cm	Ni (150 nm)	780°C −45 min	Few-layer	Tite et al., 2014
a-C(5 nm)/Ni/n-Si	KrF laser, λ = 248 nm, τ = 20 ns, ν = 10 Hz, fluence = 15 J/cm2	10^−4^ Pa	RT- 150 s	Graphite	3.6 cm	Ni (150 nm)	780°C −45 min after C deposition	Textured few layer	Tite et al., 2016
a-C (40 nm)/Ni/n-Si	KrF, λ = 248 nm, τ = 20 ns, ν = 10 Hz, fluence = 40 J/cm^2^	10^−4^ Pa	RT	Graphite	3.6 cm	Ni (300 nm)	780°C −45 min after C deposition	Self-organized multilayer	Fortgang et al., 2016
a-C/Ni/Si(111) a-C/Cu/Si(100)	Nd: YAG, λ = 355 nm, τ = 8 ns, ν = 10 Hz, fluence = 3.18 J/cm^2^	10^−5^ Torr	700, 750, 800°C 100 s	HOPG	50 mm	Ni (150-250 nm) Cu (150-250 nm)	700, 750, 800°C - 100 s during C deposition	Few- and multilayer	Kumar et al., 2017
a-C/Ni/a-C/Ni/Si(100)	Ti:sapphire, λ = 800 nm, τ = 35 ns, ν = 1 kHz, laser energy = 3.5 mJ/pulse	10^−5^ Torr	500°C	HOPG	60 mm	double Ni (100 nm)	500°C during C deposition	Large-area, Few-layer	Dong et al., 2017
Sn/a-C/SiO_2_/Si	ν = 10 Hz, laser energy = 30mJ	5.10^−5^ Pa	RT	Carbon	5 cm	Sn (500nm)	250°C, after C, Sn deposition	Multilayer	Vishwakarma et al., 2017
a-C/Cu foil	Nd: YAG, λ = 1064 nm, τ = 6 ns, ν = 5 Hz, laser energy = 50 mJ/pulse	10^−5^ Torr	300, 400, 500°C −2 and 30 min	Graphite	5 cm	Cu foil	300, 400, 500°C −2 and 30 min during C deposition	Few-layer	(Abd Elhamid A. E. M. et al., 2017)
a-C/Ni-Cu alloy	Nd:YAG, λ = 1064 nm, τ = 6 ns, ν = 10 Hz, laser energy = 150 mJ for (Ni) ν = 5 Hz, laser energy = 100 mJ for (C)	4.10^−6^ Torr	RT or 600°C for Ni deposition RT for C deposition	HOPG	5 cm	Ni-Cu alloy	600°C −30 min before C deposition	Few-layer	(Abd Elhamid A. M. et al., 2017)
a-C/Cu foil	CO_2_ laser, λ = 10.6 μm	4.10^−6^ Torr	RT	Pyrolytic Graphite	50 mm	Cu foil	RT to 700°C 15 min	Sharp folded, Wrinkled graphene	Kaushik et al., 2014
Ni/a-C:N/SiO_2_	femtosecond laser, λ = 800 nm, τ = 60 fs, ν = 1 kHz, fluence = 5 J/cm^2^	N_2_ pressure: 0.5, 1 and 10 Pa	RT	Graphite	36 mm	Ni (150 nm)	780°C −30 min after Ni deposition	Tri-layer bernal ABA configuration	Maddi et al., 2018

## PLD graphene growth without metal catalyst

Many groups have used the PLD technique to grow graphene without metal catalyst as can be seen in Table 1.

To mention just a few, Kumar and Khare (2014) demonstrated the formation of multi-layer and few-layer graphene on fused silica using Nd:YAG ultraviolet laser ablation of a graphite target under temperatures starting from RT, then 300, 500, and 700°C, without a metal catalytic layer. Raman results show the characteristic features of sp^2^ bonded carbon atoms: G band, D band and 2D band. The intensity ratio of the G and 2D bands for growth at RT is about I_2D_/I_G_ ~ 0.33 corresponding to the formation of multilayer graphene. Synthesis at 700°C gives I_2D_/I_G_ ~ 0.47 indicating the formation of few-layer graphene (<5 layers of graphene).

Another work highlighted the technique to grow few-layer graphene on non-metallic substrates using KrF excimer laser ablation of ordered pyrolytic graphite (HOPG) during different ablation times at 750°C without a metal catalyst (Xu et al., 2014). The results show the formation of the uniform few-layer graphene, with the intensity ratio of G and 2D bands I_2D_/I_G_ ~ 0.5, and an estimated crystallite size of ~38 nm. The transmittance of these samples was found to be around 95%.

By using HRTEM, the authors found the bilayer structure to be predominant in their sample (more regions show two dark lines) and the grain size was about 40 nm. This demonstrated the formation of few-layer graphene with high transmittance without a metal catalytic layer using the PLD technique at low temperature with small grain size.

In a recent study, Ren et al. (2017) grew nitrogen doped graphene (NG) *in situ* using ultraviolet pulsed laser deposition in the presence of nitrogen on Si/SiO_2_ substrates without the need for a metal catalytic layer. Different nitrogen doped graphene was grown with different nitrogen concentrations and the highest value was 3.3 at% of N. The authors clearly demonstrate the production of N-doped graphene thin film using a PLD technique, since two components “pyridinic,” and ”pyrrolic” N of N1s peak can be observed. In the same study, the authors also reported that the nitrogen-doped graphene chemically enhanced the Raman signal compared to pristine graphene and that the nitrogen content could be modulated by adjusting the nitrogen gas pressure.

In short, it is clear that the PLD way of growing graphene without a metal catalytic layer is satisfactory for direct one-step synthesis of graphene and doped graphene on different substrates. However, up to now, this technique has only enabled the production of few-layer or multi-layer graphene but not monolayer. Still, it is the only physical vapor deposition method that makes it possible to avoid the transfer process after graphene growth and is a way forward in the graphene and doped graphene synthesis field.

## PLD graphene growth using metal catalyst

Using a catalytic metal layer is one of the most widely used methods of producing graphene either by CVD and by PLD. To obtain graphene on a metal catalytic layer, free standing graphene is obtained by etching with acid and then transferring it onto another substrate of choice, which may be the most effective method. When grown on a metal layer, the resulting graphene has more uniform layers compared to mechanical exfoliation producing graphene in a wide range of thicknesses (Maddi et al., 2018). Several metals including ruthenium (Wintterlin and Bocquet, 2009), platinum (Sutter et al., 2008), nickel (Reina et al., 2009; Koh et al., 2010, 2012; Tite et al., 2016), and copper (Abd Elhamid A. E. M. et al., 2017) have been used in the synthesis of graphene.

With the PLD technique, common metals have also been used for graphene production. These include Ni, Cu, Co, and Fe that have lattice constants of about 0.352, 0.361, 0.251, and 0.287 nm, respectively (Koh et al., 2012). Among them, Ni and Cu have the smallest lattice mismatches with graphene lattice (0.357 nm). Co has the highest solubility for carbon and Fe is cheaper than Ni and Co. Today, nickel and copper are receiving the most attention as substrate materials for graphene growth because they are inexpensive and are standard materials for electronic applications. Other metals and alloys including tin (Vishwakarma et al., 2017) and Ni-Cu alloy (Abd Elhamid A. M. et al., 2017) have also been used for graphene synthesis.

PLD graphene was first produced with a metal catalytic layer in 2010 by Zhang and Feng (2010) using deposition temperatures ranging from 1,000 to 1,300°C. Subsequently, few-layer graphene films were produced by PLD at reduced temperature by Koh et al. (2010). Table 2 gives an overview of different studies on graphene synthesis using the PLD technique with a metal catalytic layer. The table shows that Ni is the most widely used catalyst in these studies. The next section is divided into two parts: (i) an overview of recent studies on graphene growth by PLD using Ni catalyst, (ii): a review of recent studies on graphene growth by PLD using other metal catalysts.

### PLD graphene growth using Ni catalyst

Nickel thin film with its high carbon solubility, low cost and ease of fabrication in electronic devices has been extensively used as metal catalyst in PLD graphene synthesis. Its high carbon solubility makes it difficult to control the number of graphene layers. Thus, in most cases, instead of single-layer graphene, either few-layer/multilayer graphene or a mixture of single-layer, bilayer and few-layer/multi-layer graphene are formed (Wang et al., 2011; Koh et al., 2012; Hemani et al., 2013). It appears that using polycrystalline nickel leads to a higher percentage of multi-layer graphene due to the presence of grain boundaries. Conversely, a large area of monolayer or bilayer graphene can be obtained on a single-crystalline nickel (Reina et al., 2009).

**Figure 7:**
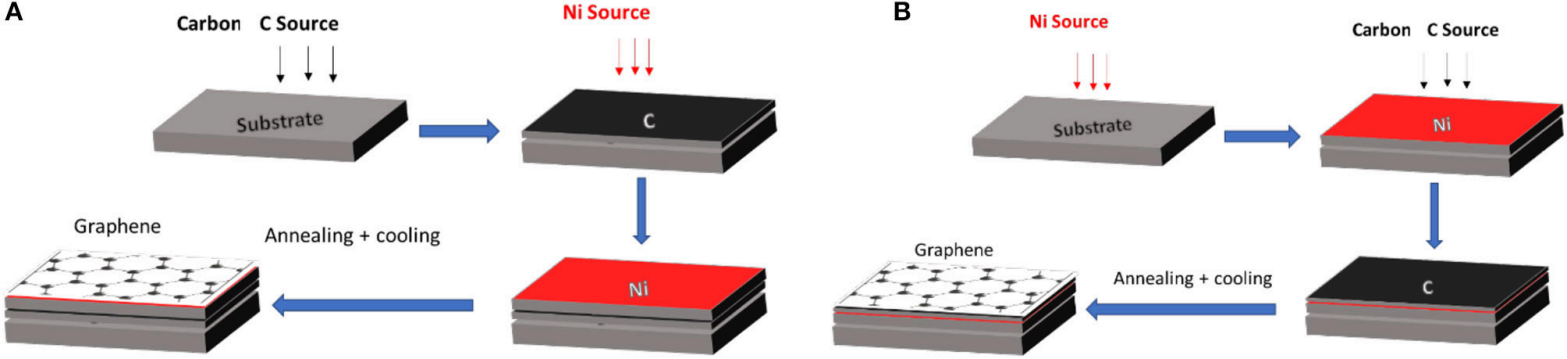
Different stacking orders using Ni on graphene growth **(A)** Sequence: Ni/a-C/substrate. **(B)** Sequence: a-C/ Ni/substrate.

A close look at the literature shows that there are two possible stacking sequences for graphene growth using polycrystalline Ni catalyst, which could be extended to other metal catalysts. These stacking configurations, Ni/a-C/substrate and a-C/Ni/substrate are shown in Figure 7. The first stacking sequence is the nickel layer on top of the carbon layer, on the substrate. The second sequence is the reverse; the carbon layer is on top of the Ni layer. While numerous metals can be used as a catalyst, nickel is probably the most promising for low temperature growth. Many parameters, including the heating rate, cooling rate, and laser power, growth time and temperature influence the quality of graphene films (Koh et al., 2010).

**Figure 8:**
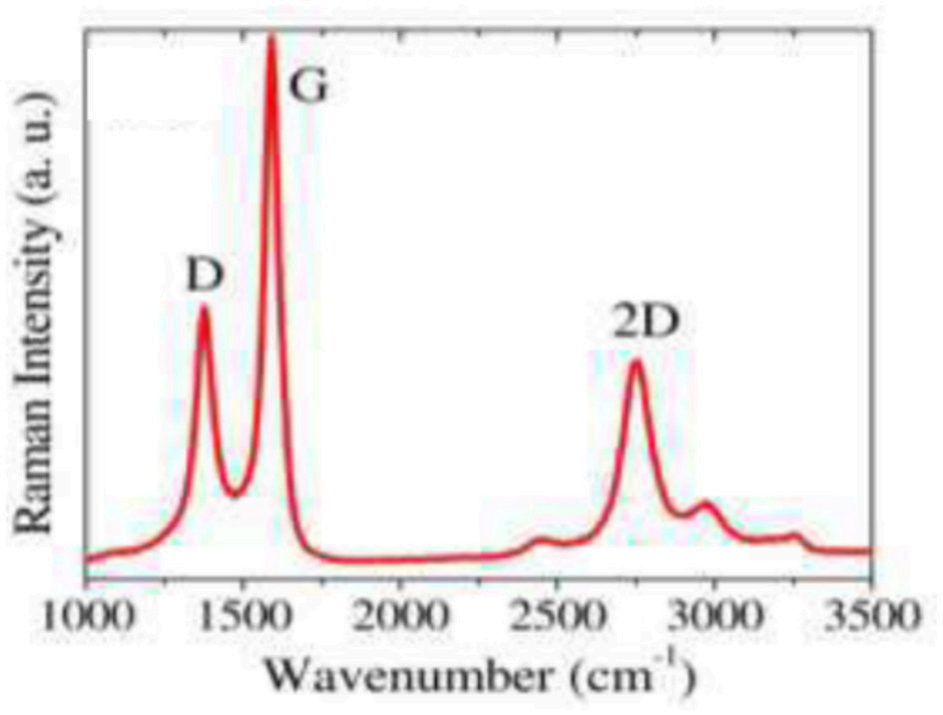
Raman spectrum at 442 nm of Ni/a-C(20 nm)/Si after thermal annealing at 780°C ([Reproduced from Tite et al. (2014) with permission of AlP Publishing].

In their study, Tite et al. (2014) reported few-layer graphene growth using the first stacking order from Ni (150 nm)/a-C (20 nm)/n-Si stacking, at a growth temperature of 780°C. Figure 8 shows the Raman spectrum of the graphene, with the well-defined D, G, 2D bands, and the intensity ratio of I_2D_/I_G_ ~ 0.4. The resulting few-layer graphene has been tested as high efficiency surface enhanced Raman scattering (SERS) active substrate for molecular diagnostics.

**Figure 9:**
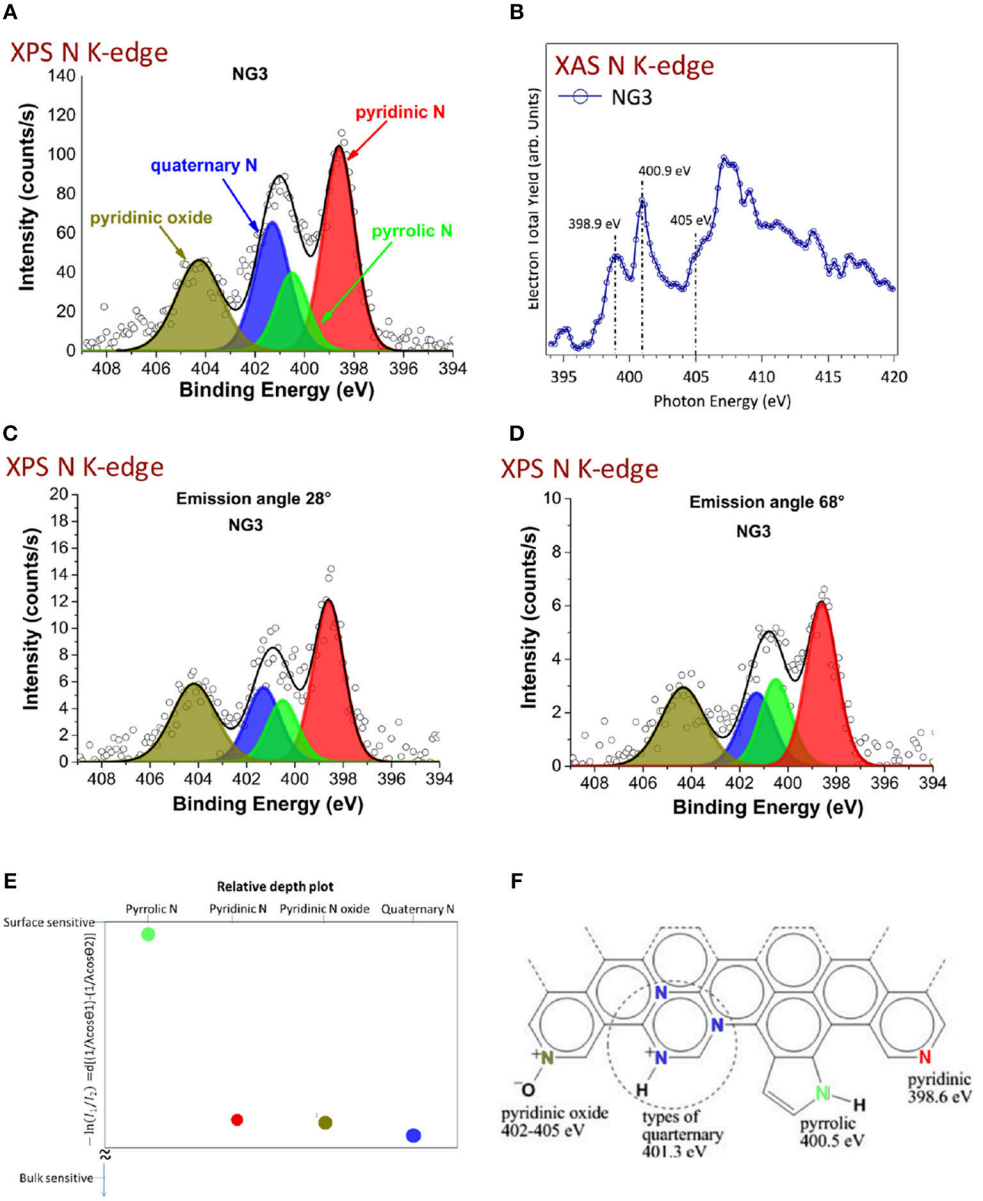
Chemical investigation of the nitrogen chemistry of the graphene layers. **(A)** XPS N K-edge of N-doped graphene (2.4%N); **(B)** XAS N K-edge of N-doped graphene; **(C,D)** ARXPS N K-edge of the N-doped graphene with an emission angle of 28 and 68°; **(E)** Ratio of intensity I1 (from ARXPS with 68°) to intensity I2 (from ARXPS with 28°) for each of the four nitrogen chemical contributions deduced from XPS, giving rise to a surface predominance of the pyrrolic from (green signal); **(F)** molecular scheme of the various N chemical forms identified by XPS and XAS in a N-doped graphene monolayer [Reproduced from Maddi et al. (2018) with permission of Nature Publishing Group].

Recently, another work (Maddi et al., 2018) reported the synthesis of trilayer nitrogen doped graphene with ABA (Bernal) configuration using the same stacking sequence. This work opens the possibility of growing nitrogen-doped graphene using the PLD method using femtosecond laser ablation of graphite under nitrogen atmosphere. In addition, the amount of nitrogen can be controlled by monitoring the nitrogen pressure. Quaternary, pyridinic, and pyridinic oxide nitrogen were found to be distributed throughout the graphene layer and the pyrrolic nitrogen was predominant in the top layer, as shown by XPS and angle resolved-x-ray photoelectron spectroscopy (ARXPS) (Figure 9).

**Figure 10:**
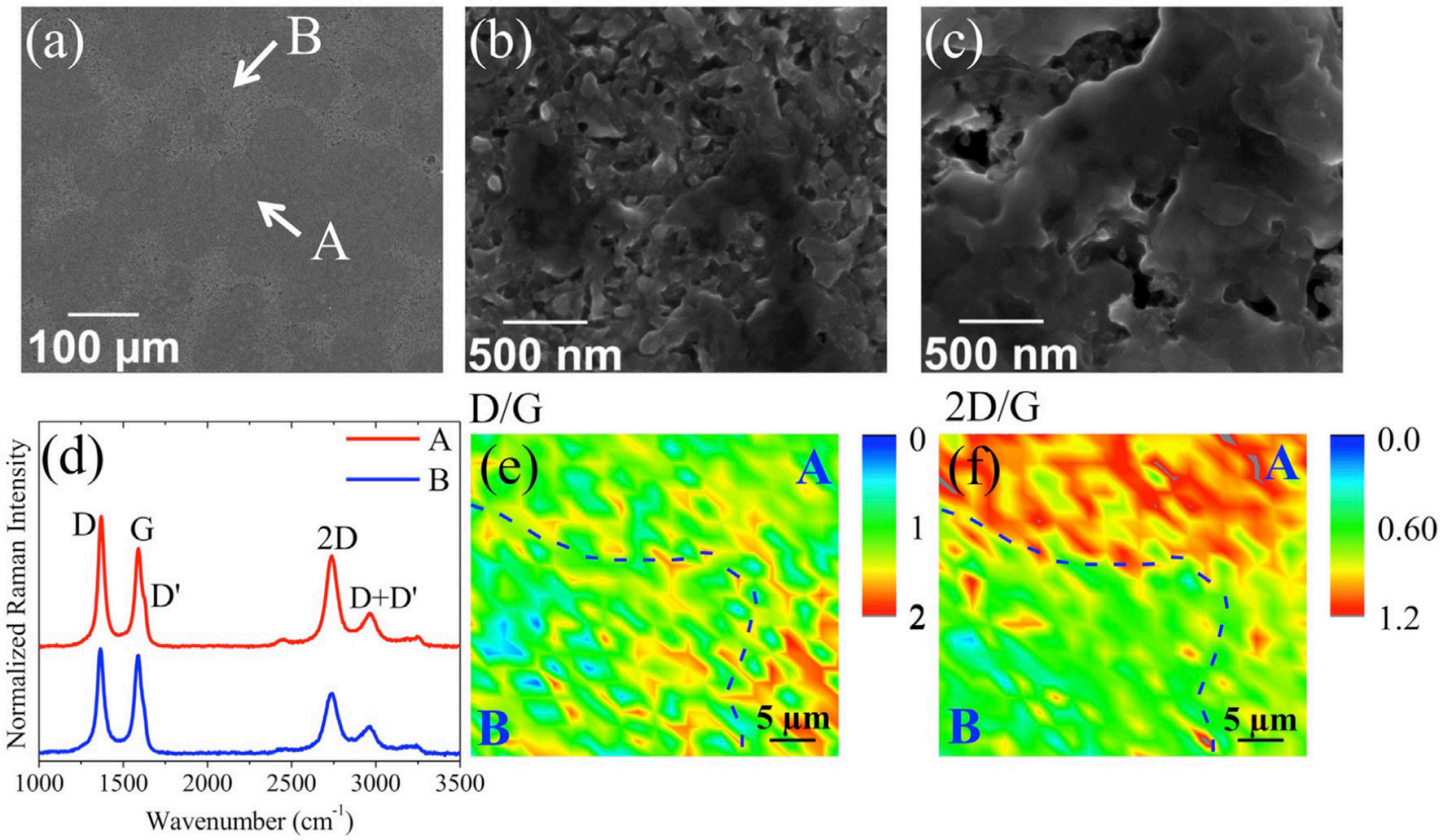
SEM images of a-C (5 nm)/Ni/Si after thermal processing at **(a)** 500 × and 12,000 × magnifications for the areas A **(b)** and B **(c)** showing a 3D porous structure; **(d)** typical Raman spectra at 442 nm taken in regions A and B. Raman mapping at 442 nm of the intensity ratio on 40 μm × 40 μm of **(e)** D/G and **(f)** 2D/G, respectively. The dashed lines are guides delimiting regions A and B [Reproduced from Tite et al. (2016) with permission of Elsevier].

In the second stacking configuration a-C/Ni/substrate, few-layer textured graphene was obtained on n-doped Si Tite et al. (2016). First, a Ni layer was deposited on Si substrate by thermal evaporation. Subsequently amorphous carbon was deposited on the Ni layer buffered Si substrate using KrF excimer laser PLD technique. The graphene was formed by conversion of the amorphous carbon film using a post-annealing process at 780°C for 45 min. Figure 10A shows the SEM image of as-grown graphene, with two distinct surface morphologies indicating the texture of the surface, labeled A and B and highlighted in Figures 10B,C respectively. Figure 10D shows some typical Raman spectra in the two aforementioned regions.

The characteristics graphene bands D, G, and 2D are well-defined and the intensity ratio I_2D_/I_G_ is in the range of ~0.6–1.2 indicating the formation of few-layer graphene in both A and B regions. Raman mapping of D/G and 2D/G (Figures 10E,F, respectively) of regions A and B confirmed that the Raman intensity of the two modes in region A is higher than in region B. The formation of few-layer graphene is clear in both regions, but the intensity is significantly higher in region A. The few-layer graphene obtained was directly used as electrode for electrochemical applications without the need for transfer.

**Figure 11:**
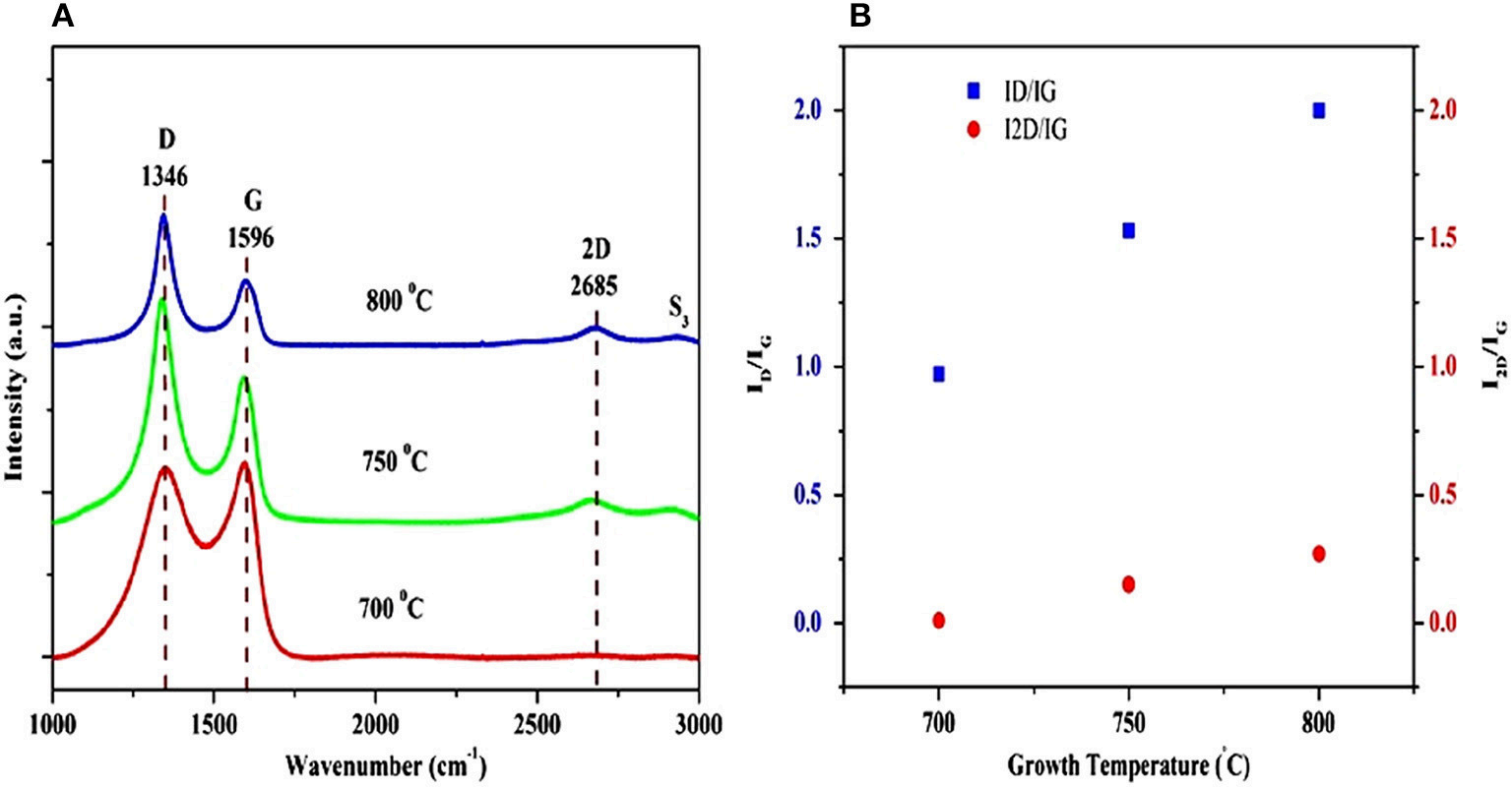
**(A)** Raman spectra of graphene films deposited on Ni/Si substrate at three different growth temperatures; **(B)** I_D_/I_G_ and I_2D_/I_G_ intensity ratios of graphene layers as a function of the growth temperature [Reproduced from Kumar et al. (2017) with permission of Springer]. **(B)** shows the I_D_/I_G_ and I_2D_/I_G_ ratios as a function of growth temperature. Clearly, the intensity ratios I_D_/I_G_ and I_2D_/I_G_ increased with the increase in growth temperature. The I_2D_/I_G_ ratio for the graphene film deposited at 750°C was 0.15, confirming the formation of multi-layer graphene (7–8 layers). The I_2D_/I_G_ ratio for graphene films grown at 800°C was 0.27, corresponding to few-layer graphene (4–5 layers).

Recently, Kumar et al. (2017) produced few- and multi-layer graphene on different substrates (Si, SiO_2_/Si, Quartz) by pulsed laser ablation of a highly ordered pyrolytic graphite target, using a pulsed nanosecond Q-switched Nd:YAG laser at 355 nm (3.5 eV) and a Ni catalytic layer. According to the authors, a few layers of graphene were deposited at a substrate temperature of 800°C, whereas multi-layer graphene was formed at a lower substrate temperature of 750°C. Therefore, the number of graphene layers was reduced while defects increased, when the growth temperature was increased. Thus, the number of graphene layer can be controlled (4–5 layers) by adjusting the growth temperature. Figure 11 (a) exhibits the Raman spectra of the graphene film grown on Ni/Si at three different growth temperatures. The 2D peak gradually disappeared with a decrease in the substrate temperature from 800 to 700°C, revealing a significant reduction in the quality of the graphene films when the growth temperature was reduced from 800 to 700°C.

**Figure 12:**
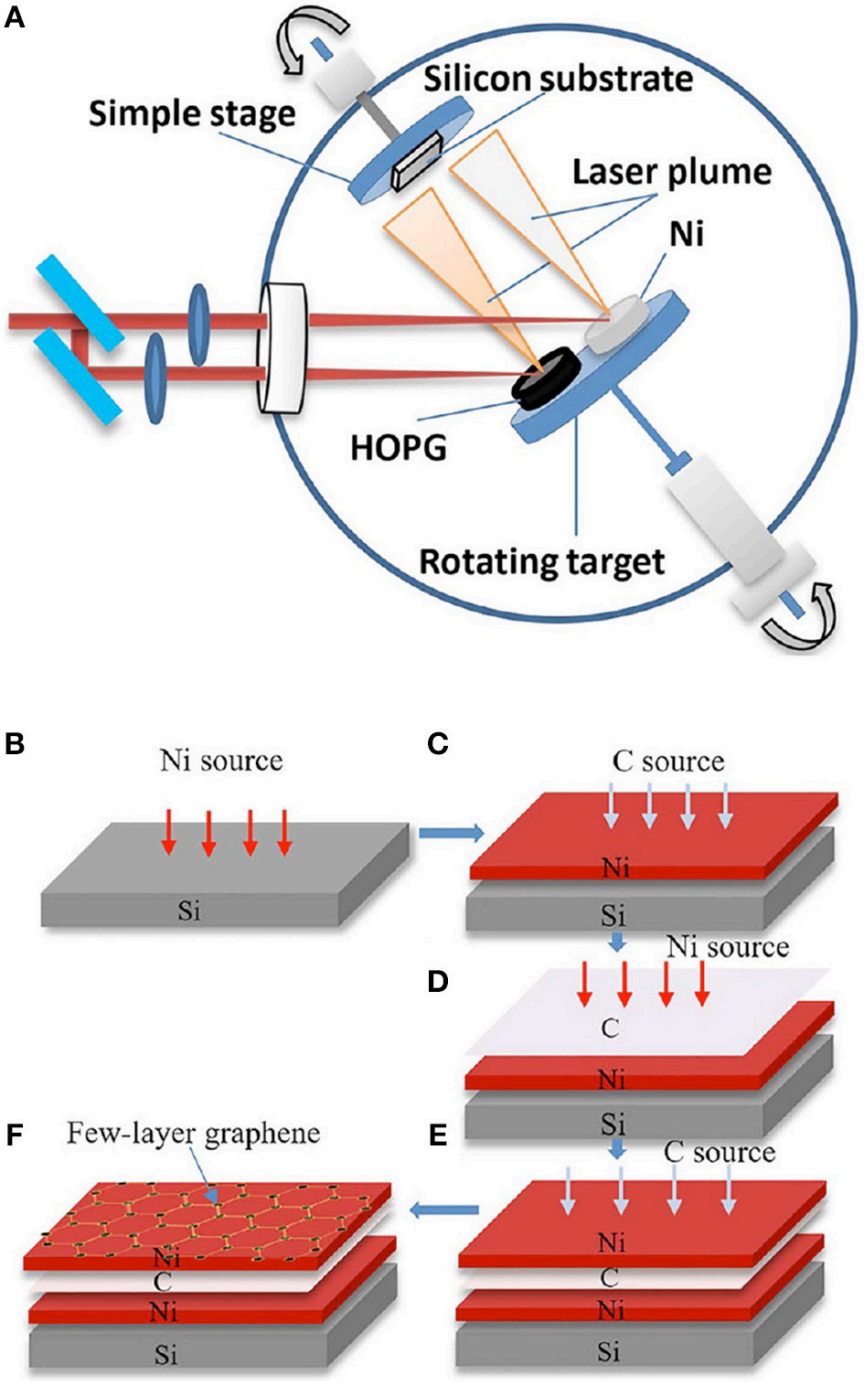
**(A)** Schematic image of the laser deposition system; steps **(B–F)** are used in few-layer graphene deposition [Reproduced from Dong et al. (2017) with permission of Springer].

In an another recent study using the a-C/Ni/substrate stacking configuration, Dong et al. (2017) demonstrated the production of large-area, few-layer graphene sheets, with few defects, by femtosecond PLD (Figure 12A) at a relatively low temperature (500°C) using a double-layer Ni catalyst. The authors claimed that using a double layer Ni catalyst makes it possible to obtain large area graphene with good electrical properties.

As illustrated in Figures 12B–F, the first Ni (100 nm) catalyst layer was deposited on silicon, and HOPG was irradiated to obtain a carbon film on the first catalyst layer (C/Ni/Si). Next, the second Ni (100 nm) catalyst layer was deposited on the carbon film. The double-layer catalyst exhibits a sandwich structure (Ni/C/Ni/Si). Last, few-layer graphene was deposited on the second Ni layer, by irradiating HOPG.

**Figure 13:**
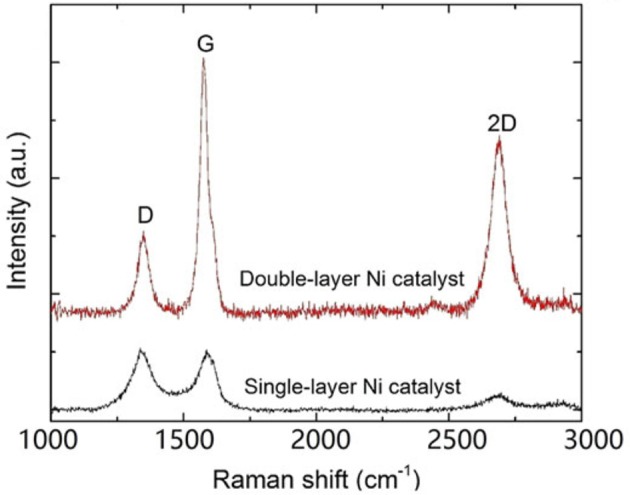
Raman spectra of samples with single- and bilayer catalysts deposited at 3 mJ [Reproduced from Dong et al. (2017) with permission of Springer].

From the Raman results in Figure 13 and Table 4, it can be seen that large area few-layer graphene was obtained on the double–layer Ni. The I_D_/I_G_ and I_2D_/I_G_ values are summarized in the table, and the I_2D_/I_G_ value of 0.69 for the film on the double-layer catalyst corresponding to 3–4 layers. The resulting samples were transferred onto SiO_2_/Si substrates and the measured electrical resistivity was as low as 0.34 mΩ.cm.

**Table 4:** Intensities and intensity ratios of D, G, and 2D peaks for films deposited on single- and bilayer Ni catalysts using laser energy of 3 mJ (Dong et al., 2017).

**Ni layer**	***I***_D_ **(a.u.)**	***I***_G_ **(a.u.)**	***I***_2D_ **(a.u.)**	***I***_D_/*I***_G_**	***I***_2D_/*I***_G_**
1	506	493	137	1.03	0.28
2	692	2,194	1,524	0.32	0.69

To sum up, graphene has been grown by many groups using the PLD technique with a nickel catalytic layer and two stacking orders: Ni/a-C/substrate and **a**-C/Ni/substrate. In contrast, few studies have been performed using PLD for the first stacking configuration Ni/a-C/substrate. However, using this order makes it possible to elucidate the diffusion mechanism of carbon in nickel during the formation of graphene. For instance, Weatherup et al. (2013) used this stacking order to demonstrate the graphene growth mechanism by *in situ* XPS during annealing at 600°C in vacuum. These authors identified four C1s components in the spectra at ~ 283.2 eV (C_A_), ~ 283.8 eV (C_Dis_), ~ 284.4 eV (C_Gr_), and ~ 284.8 eV (C_B_). The C_A_ and C_Dis_ components appears first during heating, and were assigned to carbon bound to Ni surface sites, and interstitial carbon dissolved in the Ni catalyst, respectively. C_Gr_ and C_B_ peaks appeared simultaneously some time later, corresponding to the formation of graphitic carbon at the catalyst surface and the disordered sp^3^ carbon, respectively. For the Ni 2p_3/2_ core level spectra, two main components Ni_M_ (metallic Ni) and Ni_Dis_ (interstitial solid solution of C in Ni) were detected. The metallic Ni is more surface sensitive and the interstitial solid solution of C in Ni is bulk sensitive.

Conversely, many studies have been performed on the second stacking order for graphene growth. One of the main difference between the two stacking orders is that with the second configuration (a-C/Ni/ substrate), technically, the Ni catalyst can be heated while depositing the solid carbon source. This procedure can increase the nickel grain size and consequently enlarge the grain size of the resulting graphene. However, the difference in the formation, and the type and quality of the graphene between these two stacking orders requires further research.

According to the studies summarized in Table 3, the quality of the grown graphene depends on the thickness of Ni catalytic layer, the thickness of the carbon layer, the deposition temperature and duration, the annealing time and temperature, and the cooling rate. All these parameters, plus those of the PLD technique, e.g., fluence, laser wavelength, and the repetition rate, influence the quality of the graphene. Moreover, because of the high solubility of carbon in nickel, controlling the number of layers of the grown graphene remains a challenge in graphene synthesis using polycrystalline nickel as catalyst.

It is worth mentioning that the production of graphene using both stacking orders and Ni catalyst has been achieved with other methods such as thermal annealing (Lee et al., 2016), filtered cathodic vacuum arc (FCVA) (Zheng et al., 2010; Seo et al., 2012), rapid thermal processing (RTP) (Chu et al., 2012; Pan et al., 2013; Xiong et al., 2013), plasma enhanced chemical vapor deposition (PECVD) (Delamoreanu et al., 2014), pulse arc plasma deposition (PAPD) (Fujita et al., 2012; Banno et al., 2013; Miyoshi et al., 2017) and radiofrequency (RF) magnetron sputtering (An et al., 2016).

### PLD graphene growth using other metal catalysts

Apart from nickel, other metals catalysts are used for graphene synthesis with the PLD method. Graphene growth using copper (Cu), cobalt (Co), tin (Sn), iron (Fe), and Cu-Ni alloys as catalysts has also been reported.

Koh et al. (2012) published an interesting comparison of PLD graphene synthesis with Ni, Co, Cu, and Fe metal catalysts. These authors demonstrated that under controlled cooling conditions: initial cooling rate of 1°C/min to 550°C, followed by a faster cooling rate of 20°C/min to room temperature, few-layer graphene formed on nickel, but not on the other metal catalysts. When the cooling rate was increased, graphene also formed on Co and is much more homogeneous than on Ni. It was therefore concluded that the cooling rate is an important parameter in growing graphene. The authors explained that because carbon is highly soluble in cobalt with a low diffusion coefficient, when the cooling rate is slow, carbon atoms can take longer to diffuse further within the cobalt, whereas, with a high cooling rate, the saturated carbon near the surface is maintained, thereby allowing adequate precipitation that enables the formation of few-layer graphene. With the Ni catalyst, which is almost half as soluble in carbon than Co, carbon can easily exceed the solubility limit when the temperature is reduced gradually. The same authors observed that different cooling rates strongly influence the graphene growth with Ni catalyst. At a moderate cooling rate of 50°C/min, few-layer graphene formed, whereas at a faster cooling rate of 100°C/min, no graphene was observed.

Using continuous wave CO_2_ laser ablation of pyrolytic graphite target placed in a vacuum chamber at a pressure of 10^−6^ Torr, Kaushik et al. (2014) observed graphene ribbons synthesis. The graphene films were deposited on Cu foils kept at different temperatures, and raising the temperature of Cu to 700°C led to the formation of large surface grains. Nanostructures of graphene on Cu foil were observed at low temperature about 400°C. On the same Cu foil substrate, (Abd Elhamid A. E. M. et al., 2017) demonstrated the possibility of growing graphene using the PLD technique at the relatively low temperature of 500°C and an optimal cooling rate.

Vishwakarma et al. (2017) reported an attempt to grow multi-layer graphene at a low temperature (250°C) using another metal catalyst, tin (Sn) and the PLD technique. In fact, this is the first attempt to grow PLD graphene using tin as catalyst. The authors suggest that the resulting graphene is still of micrometer order, but this approach could open a new route for free graphene growth at low temperatures.

Another attempt was made by Elhamid et al. on metal Ni-Cu composite substrates (Abd Elhamid A. M. et al., 2017). These authors reported that graphene synthesis can be achieved through graphite ablation using the PLD technique on catalyst Ni-Cu composite substrates at room temperature. The intensity ratio of the 2D and G Raman bands was 0.66, indicating the formation of tri-layer graphene. Nickel-copper substrates exhibit excellent graphene growth ability at room temperature, which was attributed to the intrinsic spinodal surface structure favoring diffusion in grain boundaries.

This close look at the literature, confirms that few studies have been conducted on graphene synthesis using catalytic metals other than nickel using the PLD technique. Further investigation of graphene growth on Cu, Fe, Co, Sn, and Ni-Cu using PLD is thus needed. Such studies would advance our understanding of the optimal growth conditions for graphene production using other metal catalysts and PLD.

To conclude this section, one can say that using the PLD technique with a Ni metal catalytic layer has generally enabled the formation of few-layer graphene like the method without metal catalytic layer. The difference between the two techniques is that the one with a catalytic layer can produce a more uniform, larger area graphene than the one without metal catalyst. However, by optimizing the growth conditions with Ni catalyst, it is possible to produce single layer graphene using PLD. One of the advantages of the PLD method without metal catalyst is that one can grow few-layer or multi-layer graphene directly on the desired substrate, which eliminates the need for transfer. The two possible stacking orders in the PLD technique using a metal catalyst could lead to graphene synthesis. In the stacking order without the metal catalyst on top of the amorphous carbon layer, graphene forms between the metal and the substrate after thermal annealing. Conversely, in the other stacking order, the amorphous carbon is on top of the metal catalyst, and graphene forms on top of the metal layer after thermal annealing. Nickel is still the most widely used catalyst in PLD graphene synthesis today. Even so, PLD graphene production using nickel catalyst need to be optimized by playing with the Ni/a-C thickness ratio, annealing conditions such as time, temperature, heating and cooling, and other metals catalysts are needed to open new routes for graphene fabrication.

## Applications of graphene grown with the PLD technique

Up to now, few works have reported applications for graphene produced using PLD. In fact, potential applications range from p-n junction for electronic and optoelectronic applications to sensing function for electrochemistry and transparent electrodes.

Sarath Kumar et al. (2013) manufactured PLD graphene films in p–n junctions devices. The graphene film was deposited on SiNx/Si substrates with argon or nitrogen background gases. The graphene grown in argon gives p-type conduction whereas the graphene grown under nitrogen environment exhibits n-type semiconducting behavior that results from the nitrogen doping during the deposition of the film. The p–n junction diodes based on the resulting graphene were diode-like, which was attributed to an increased difference in the Seebeck coefficient of p- and n-type films. The good conductivity of the PLD graphene makes it promising for field emission (FE) and sensor applications. With PLD, few-layer graphene with a sharp folded nanostructure was deposited on copper (Cu) substrates (Kaushik et al., 2014). After being transferred to a SiO_2_/Si substrate, the graphene nanoribbon showed good FE performance with a turn-on field of 1.4 V/μm, due to a large number of emission sites in the sharp edges.

**Figure 14:**
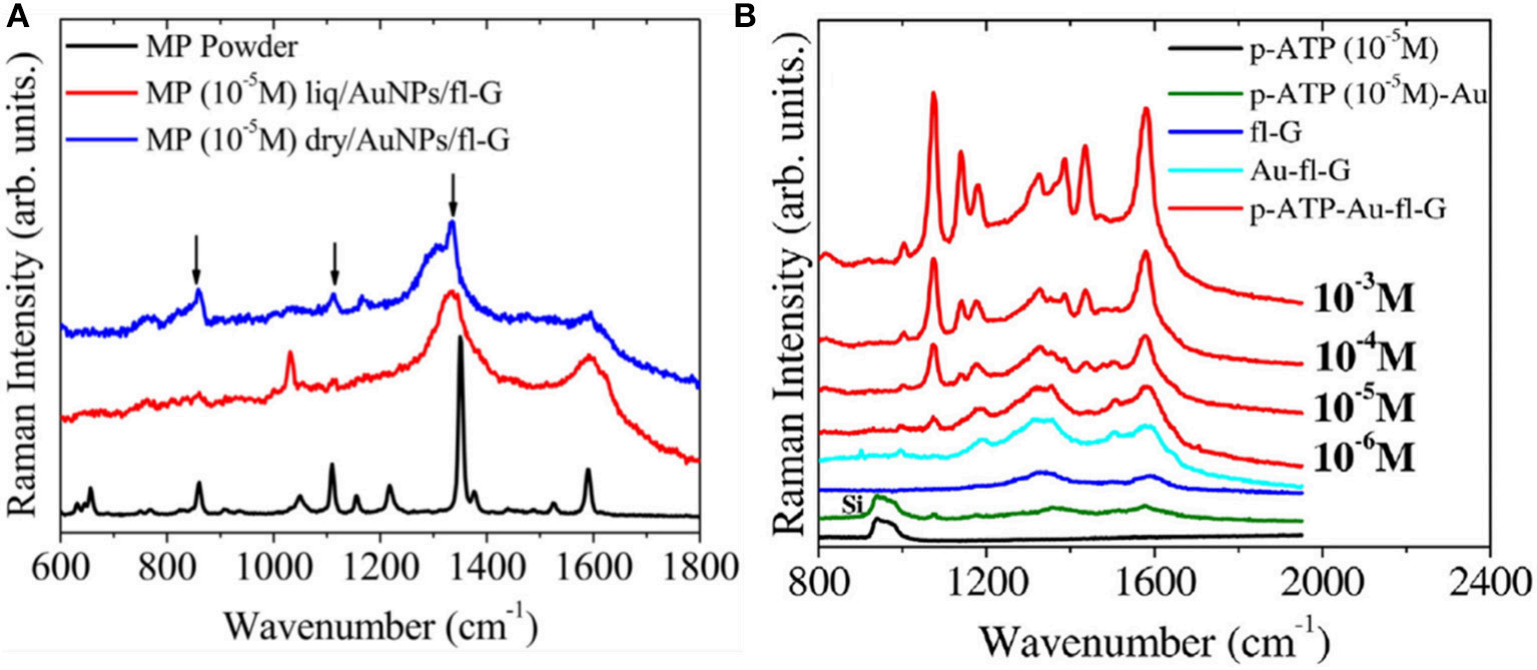
**(A)** Raman spectra at 633 nm of methyl parathion (MP) on Au-fl-G concentrated at 10–5 M and 10–4 M at liquid and dry states. **(B)** Raman spectra at 633 nm of p-ATP(10–5 M) on Si, p-ATP(10–5 M) on AuNPs deposited on Si, fl-graphene, AuNPs/fl-G, and p-ATP on AuNPs/fl-G at different p-ATP concentration (10–6 M, 10–5 M, 10–4 M, 10–3 M) [Reproduced from Tite et al. (2016) with permission of Elsevier].

Because of its high adsorption capacity, graphene has also been widely used for environmental clean-up. Recently, Tite et al. (2014, 2016) used PLD graphene to detect para-aminothiophenol (p-ATP) and methyl parathion (MP), active molecules in commercial insecticides. PLD few-layer graphene was obtained by post-annealing amorphous carbon film directly on Si substrate. Gold nanoparticles were placed on the graphene surface to improve sensitivity. As a result, the Raman spectra (Figure 14) features graphene peaks with observably enhanced intensity in the presence of increasing p-aminothiophenol (p-ATP) and methyl parathion (MP) concentrations, indicating that the insecticide can be detected at concentrations below the cytotoxic dose.

The authors suggested that the Au NPs decorated with the PLD few layer graphene substrate will become a practical and powerful platform for the harvesting the SERS (Surface enhanced Raman spectroscopy) signals of molecules, such as pesticides or pollutants for environmental safety.

**Figure 15:**
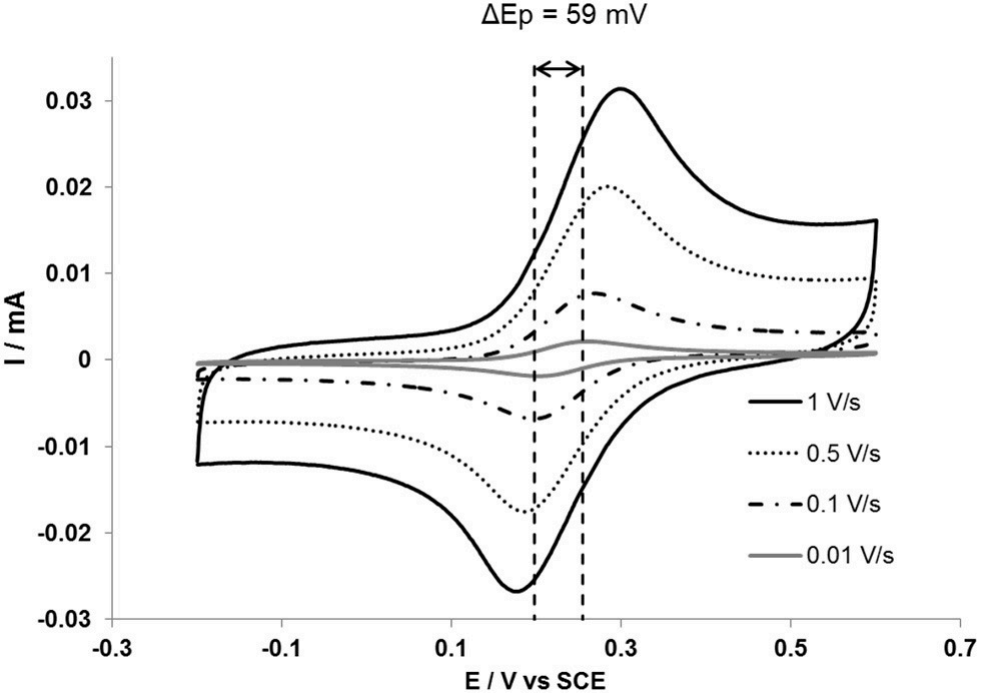
Cyclic voltammograms of a 3D self-organized graphene of 0.5 mM ferrocene dimethanol solution in 0.1 M NaClO4 aqueous electrolyte. The two vertical dashed lines help read the theoretical ΔEp value of 59 mV (Reproduced from Fortgang et al. (2016) with permission of ACS Publications].

Fortgang et al. (2016) reported a self-organized three-dimensional (3D) graphene electrode processed by PLD with thermal annealing and electrochemical testing. These authors showed that self-organized 3D graphene can be used as electrodes for various sensing functionalities. Graphene films synthetized from annealed a-C (40 nm)/Ni (300 nm)/n-Si stacking were investigated for use as high performance electrode for electrochemical applications. Cyclic voltammetry (Figure 15) revealed excellent quasi-reversible performances in electrochemical kinetics: the electron transfer of a ferrocene dimethanol (Fc(CH_2_OH)_2_) redox probe on the graphene film was shown to be reversible (ΔEp = 59 mV and Ip/Ia = 1) at scan rates below 20 mV/s for few-layer graphene. At higher scan rates, electron transfer kinetics becomes quasi-reversible (ΔEp >59 mV and Ip/Ia = 1).

**Figure 16:**
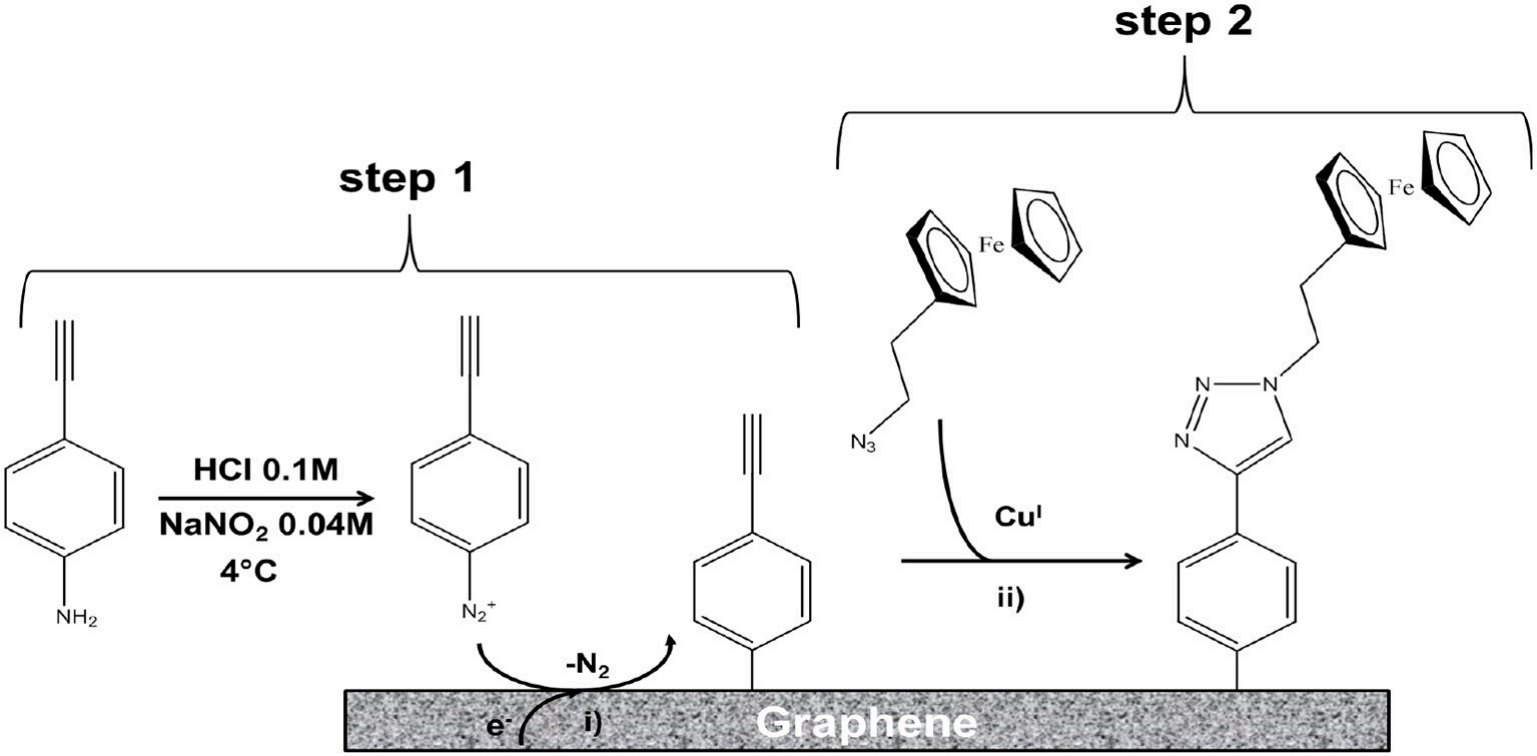
Illustration of the two-step functionalization of a 3D graphene electrode: (Step 1) Electrochemical reduction through cyclic voltammetry of *in situ* generated diazonium salts for covalent immobilization of 4-ethynylphenyl moieties; (Step 2) CuI-catalyzed Huisgen 1,3-dipolar cycloaddition between the immobilized alkyne functions and the ferrocene derivatives bearing the corresponding azide group; CuI was obtained by reducing CuII with ascorbic acid [Reproduced from Fortgang et al. (2016) with permission of ACS Publications].

The ethynyl aryl groups were shown to be successfully and robustly attached to the graphene surface, paving the way for the specific attachment of molecules bearing an azide function using the click reaction. The method was applied to ferrocene-azide to model the grafting of redox molecules on the graphene layer (Figure 16).

The electrochemical response of the functionalized electrode was studied. The authors confirmed that this addressing method was efficiently controlled by electrochemistry, showing high loading of ferrocene and a stable electrochemical response of the electrode over a period of 22 days. The quantity of the grafted molecules was higher than on other frequently used substrates such as glassy carbon or BDD due to a multi-layer grafting structure. High loading of recognition elements on the electrode should thus be possible. This method allows electrochemically controlled functionalization for addressing probes on a multielectrode device. This work opens highly promising perspectives for the development of self-organized 3D graphene electrodes with different sensing functionalities and can be applied to fragile sensing objects such as biomolecules or living systems.

In the transparent electrode field, Xu et al. (2014) demonstrated the formation of graphene using the high transmittance PLD technique: 93% for three-layer graphene, 95% for bi-layer graphene and 91% for four layer graphene. The authors observed low resistance for graphene with 2–3 layers (120, 90 s) about Rs ~ 1000 Ω ⋎-1 and high resistance over 10 kΩ ⋎-1 for graphene film obtained with 30 s of laser ablation. The authors suggested that graphene film with high transmittance and low resistance would be ideal for transparent electrodes.

## Conclusion and challenges

Graphene has remarkable properties that promise a wide range of possible applications including electronic devices, supercapacitors, batteries, composites, flexible transparent displays, solar cells, and sensors. In order to achieve the full potential of graphene material, samples of graphene of high quality, uniform morphology and large area are required. The PLD technique is appealing for the growth of graphene materials as an alternative to the conventional CVD technique. This review provides an overview of the synthesis of graphene and doped graphene deposited using the PLD technique and some potential applications. Based on the results reported here, PLD graphene of different types and of different quality, from high to low, from single to multilayer can be produced, depending on the specific growth conditions, including substrate temperature, the energy density of the irradiated laser on the target, the background pressure, annealing rate, and time invested. In addition, compared to the graphene obtained by other commonly used methods, such as mechanical exfoliation, chemical and liquid exfoliation, CVD, PLD graphene growth has the advantage of good adhesion to the substrates, rapid speed of growth and relatively low production temperature. However, PLD single layer graphene has not been obtained so far. PLD graphene is sometimes a mixture of single, bilayer, trilayer, few-layer, and multi-layer graphene. The growth conditions consequently need to be optimized in order to combine high quality with large area graphene and single layer graphene.

Compared with the extensive studies on CVD graphene materials, the use of PLD graphene is quite recent and requires a more detailed understanding of the mechanisms involved and of the optimal deposition conditions. Some of the challenges that remain are listed below:

- The synthesis of PLD graphene requires a relatively long period to optimize the deposition conditions because the fundamental mechanism of growth is not yet fully understood.
- Up to now, PLD graphene using metal catalyst is mostly prepared with Ni catalyst. Few studies report PLD graphene growth using other metal catalysts. Graphene growth on the other catalyst e.g., Cu, Co, Fe, Ag, Ni-Cu using PLD is thus worth exploring. In addition, using a metal catalytic layer in graphene growth represents an additional key parameter: the ratio of the thickness of the metal layer to that of the carbon layer. This ratio depends on the metal used and needs to be optimized in order to produce high quality graphene.
- Doped graphene can be produced using PLD. Nevertheless, up to now, only two studies have reported on nitrogen-doped graphene (n doping) and none on boron-doped graphene (p doping) using PLD. This could be a promising direction to study the effects of doping on PLD graphene in the future.
- From the point of view of the type of laser, many studies have reported PLD graphene growth using excimer nanosecond laser, while only a few studies report on femtosecond laser. It would thus be interesting to compare PLD graphene obtained using nanosecond laser and femtosecond laser, since laser directly influences the nature of the carbon-based film used as precursor of the graphene film.

Lastly, the synthesis of graphene is fundamental for all studies in terms of property and applications. Simple methods are still needed to easily control crystallinity, grain size, grain boundaries, layers and output, and high-fidelity transfer. In the past, PLD has proven to be a powerful tool to deposit a single layer of thin film. Today, it is believed that PLD will play an important role in making graphene and doped graphene materials for various applications in the future. At the same time, understanding the growth mechanism needed to produce high quality, uniform, large area graphene using PLD is indispensable. Since it is clear that no single parameter is responsible for large-area, high quality graphene, all the growth conditions need to be optimized to achieve the same or better quality graphene than that grown using CVD or mechanical exfoliation techniques.

## Author contributions

YB wrote the main part of the proof. FB, TT, CM, and A-SL have performed many experiments described in the proof. CD and FG supervised the experimental work and have completed the proof.

### Conflict of interest statement

The authors declare that the research was conducted in the absence of any commercial or financial relationships that could be construed as a potential conflict of interest.
